# A case of culture-proven cutaneous atypical mycobacterial infection caused by *Mycobacterium immunogenum*

**DOI:** 10.1016/j.jdcr.2025.02.021

**Published:** 2025-03-10

**Authors:** Faraz Yousefian, Aysham Chaudry, Francesca Ceci, Charay Jennings-Dover, Dionne Louis

**Affiliations:** a Department of Dermatology and Mohs Surgery, Philadelphia College of Osteopathic Medicine, Roswell, Georgia; b Goodman Dermatology, Roswell, Georgia; c Center for Clinical and Cosmetic Research, Aventura, Florida; d SkinPath Solutions, Smyrna, Georgia

**Keywords:** cutaneous atypical mycobacterium, infectious, *Mycobacterium immunogenum*

## Introduction

*Mycobacterium immunogenum* is an atypical, rapidly growing mycobacterium related to the *M chelonae*-*M abscessus* group. Infection with this pathogen can cause a variety of clinical manifestations, with cutaneous involvement being particularly common. Cutaneous infections include folliculitis, ulceration due to exposure to contaminated water, and postsurgical and postinjection skin infections.^1^ Additionally, *M immunogenum* may also present as erythematous papules, nodules, and plaques, often following invasive procedures.^2^, ^3^, ^4^, ^5^ Beyond the skin, it is most commonly implicated in hypersensitivity pneumonitis, associated with metalworking fluids.There have also been reports of keratitis, tenosynovitis, and cerebral abscess caused by the pathogen.^1^ Here we present a case of cutaneous nontuberculous atypical mycobacterial infection caused by *M immunogenum* in a patient with no identifiable risk factors, highlighting an unusual presentation of this pathogen.

## Case presentation

A 73-year-old Caucasian female with a past medical history of coronary artery disease, chronic kidney dysfunction, hyperlipidemia, and diabetes presented to our outpatient dermatology clinic for easy bruising of the upper and lower extremities for 2 years. She denied any past medical or family history of bleeding and autoimmune disorders. Review of systems was noncontributory. She denied any recent travel, recent trauma, or sick contacts. Her current medications included amlodipine, aspirin, chlorthalidone, ezetimibe, and empagliflozin.

**Fig 1:**
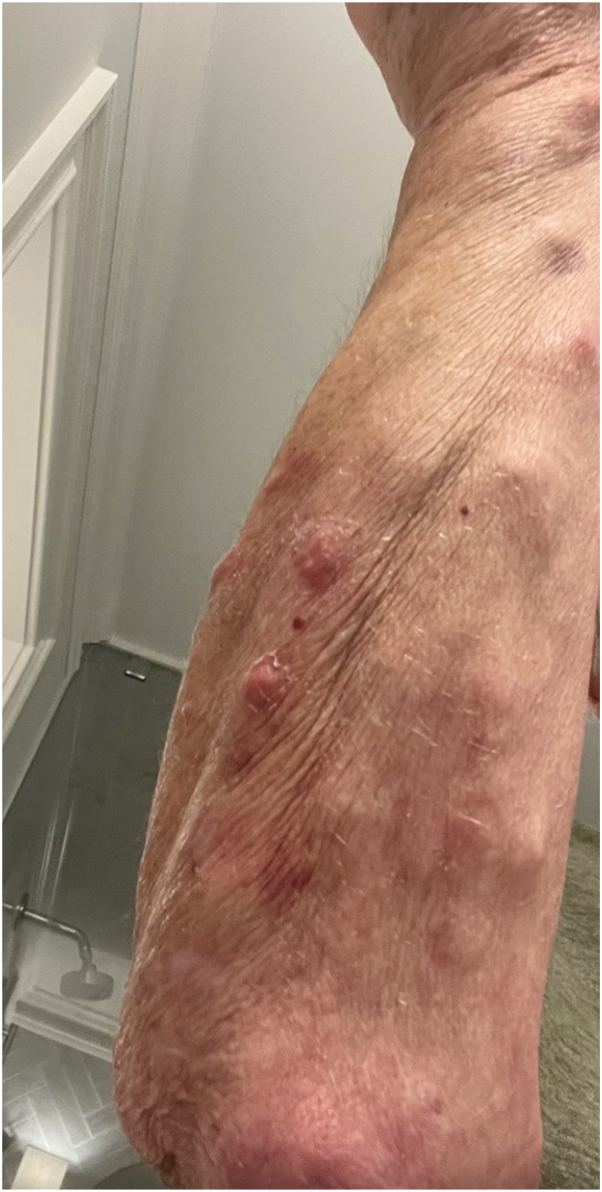
Physical examination revealing violaceous patches distributed on left upper extremity and two 1 cm nontender nodules on the upper arms.

**Fig 2:**
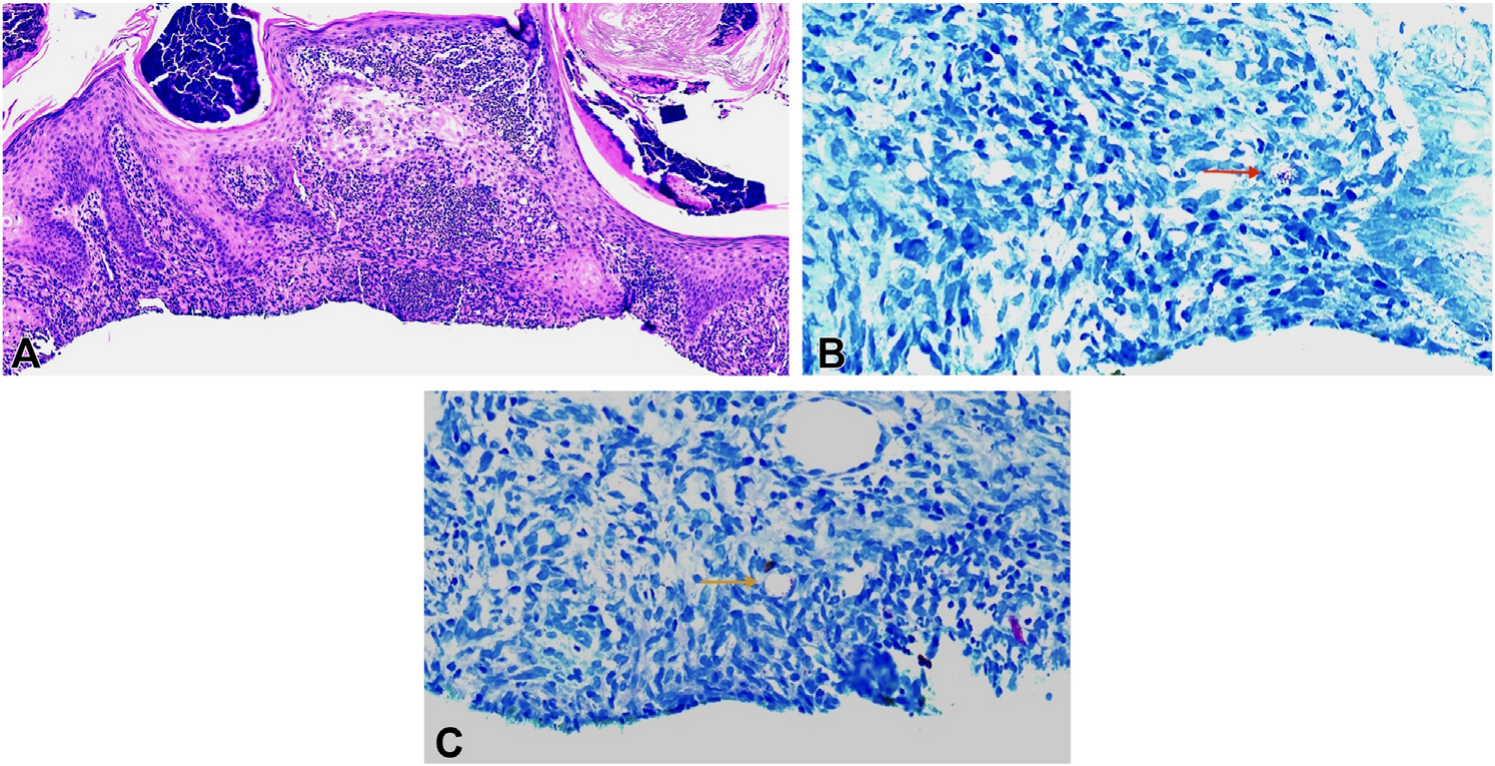
Microscopic results. **A,** A hematoxylin & eosin (H&E) stain (100×) demonstrates a suppurative and granulomatous inflammatory pattern with overlying hyperkeratotic crust, epidermal hyperplasia, a mixed inflammatory infiltrate of histiocytes, neutrophils, and lymphocytes. **B,** An acid-fast bacillus stain (400×). **C,** Fite stain (400×) both demonstrate clusters of intracellular and extracellular acid-fast bacilli (*arrows*).

**Fig 3:**
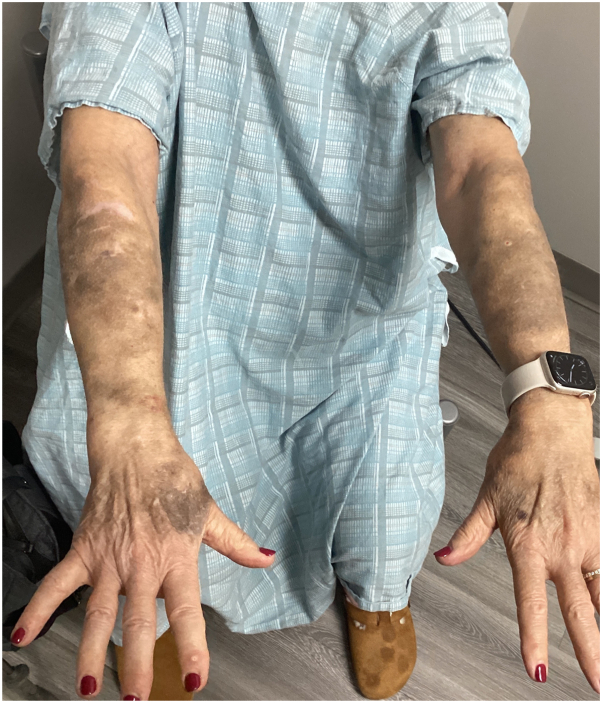
Physical examination revealing improvement on left upper extremity post-treatment.

Physical examination revealed violaceous patches on bilateral upper and lower extremities, which were consistent with senile purpura. Additionally, two 1 centimeter nontender nodules were incidentally found on the upper arm (Fig 1). Two punch biopsies of the nodules revealed suppurative and granulomatous dermatitis that was suggestive of an infection by atypical mycobacteria (Fig 2, *A*). Consequently, two acid-fast bacilli cultures and Fite stains were performed from the biopsy tissue with eventual confirmation and identification of *M immunogenum* (Fig 2, *B* and *C*). Serum testing for HIV was negative. The patient was referred to infectious disease and started on a regimen of intravenous amikacin, intravenous omadacycline, and oral azithromycin with initial improvement of the lesions within 2 months (Fig 3). The patient then developed hearing and vision problems secondary to amikacin, at which point the medication was discontinued. The patient was then started on linezolid but developed diarrhea and mild abdominal pain and this was also discontinued. Currently, the patient is tolerating oral azithromycin 500 mg and oral omadacycline 300 mg with a slow improvement of the lesions. The patient has been advised to continue the current regimen for a total duration of 12 months.

## Discussion

*M immunogenum* is a recently discovered pathogen that shares phenotypic and genotypic characteristics with *M chelonae* and *M abscessus*. They are rapidly growing, atypical mycobacteria that commonly cause waterborne nosocomial outbreaks and hypersensitivity pneumonitis associated with metalworking fluid due to their ability to create biofilms.^1^ Like these atypical mycobacteria, *M immunogenum* has also been reported to cause hypersensitivity pneumonitis in association with metalworking fluid. There have also been reports of keratitis, tenosynovitis, and cerebral abscess with *M immunogenum* infection.^2^^,^^4^

There has been an increasing incidence of cutaneous nontuberculous mycobacterial infection caused by *M immunogenum* (Table I). Majority of these cases have been described in the postprocedural setting or in the setting of immunosuppression. In one study, 28 patients developed skin lesions at the injection site after mesotherapy.^5^ There have been at least two cases of postsurgical wound infection caused by surgical material contamination. There was also a case of a tattoo infection caused by a contaminated tattoo reservoir.^4^ One case report describes disseminated infection from *M immunogenum* in the setting of immunosuppression in a renal transplant patient with cutaneous manifestations.^6^ Other reported cases include skin ulceration with exposure to contaminated water and a single case of folliculitis.^3^^,^^4^ Our case differs because this patient did not have any invasive surgeries or injections prior to disease manifestation. The patient also did not have any known risk factors such as exposure to contaminated water or immunosuppression. Only one other case has been described that is similar to ours, with presentation of erythematous papules and pustules of the abdomen and successful treatment with clarithromycin.^4^

**Table I:** Cases of *M immunogenum*

Infection setting	Description
Postprocedural	28 patients developed skin lesions at the injection site following mesotherapy
Surgical material contamination	Two cases of postsurgical wound infections due to contaminated surgical material
Tattoo infection	One case of a tattoo infection linked to a contaminated tattoo ink reservoir
Immunosuppression	One case of disseminated infection in an immunosuppressed renal transplant patient with cutaneous manifestations
Contaminated water exposure	Skin ulceration following exposure to contaminated water
Folliculitis	Single reported case of folliculitis associated with *M immunogenum* exposure

Treatment of *M immunogenum* is challenging due to the lack of clinical guidelines or a standardized approach for treatment attributed to ill-defined antimicrobial susceptibility patterns. Previously discussed cases were treated largely with clarithromycin and a possible addition of a fluoroquinolone or amikacin with successful resolution of lesions.^4^ Although limited, some information regarding susceptibility patterns exists and can be used to guide management until susceptibilities become available.^7^ Further research and a standardized approach to treatment are warranted to address the challenges posed by newly discovered bacterial species like *M immunogenum* in guiding medical management effectively.

## Conflicts of interest

None disclosed.
